# Effects of Cooking Processes on Protein Nutritional Values and Volatile Flavor Substances of Silver Carp (*Hypophthalmichthys molitrix*)

**DOI:** 10.3390/foods12173169

**Published:** 2023-08-23

**Authors:** Yin Zhang, Pengcheng Zhang, Haichuan Peng, Qiuyue Chen, Xiaolei Jiao, Jianlin Jia, Zhongli Pan, Jie Cheng, Linguo Wang

**Affiliations:** 1 Meat Processing Key Laboratory of Sichuan Province, Chengdu University, Chengdu 610106, China; zhangpc46@163.com (P.Z.); phc960205@163.com (H.P.); 18782151347@163.com (Q.C.); 19982048990@163.com (J.J.); chengjie@cdu.edu.cn (J.C.); 5196956065@163.com (L.W.); 2 Neijiang Academy of Agricultural Sciences, Neijiang 641099, China; jiao401lei@126.com; 3 Department of Biological and Agricultural Engineering, University of California, One Shields Avenue, Davis, CA 95616, USA; zlpan@ucdavis.edu

**Keywords:** silver carp, basic nutrients, gas chromatography–mass spectrometry, flavor compounds

## Abstract

Silver carp (*Hypophthalmichthys molitrix*) is normally cooked at a high temperature. In order to explore the effects of cooking methods on the protein nutritional values and volatile flavor substances of silver carp meat, the meat was fried, roasted and steamed, and its contents were determined in relation to moisture, protein, fat, ash, amino acids and volatile flavor substances. The results show that the moisture content of cooked silver carp can be ranked as follows: raw > steamed > roasted > fried. The protein content order of the four samples can be ranked: fried > roasted > raw > steamed. The ash and the fat contents of the four samples showed similar significant (*p* < 0.05) differences, in the order of fried > roasted > steamed > raw. The contents of all the amino acids in the fried meat were significantly (*p* < 0.05) higher than the contents in others, and the frying, roasting and steaming processes improved the umami taste (supported by the increased content of glutamate and aspartate) and the protein nutritional value (supported by the amino acid score, essential amino acid index and protein efficiency ratio) of the silver carp meat. A total of 51 compounds were detected in the fried, roasted and steamed meat. Frying and roasting reduced the fishy flavor of this meat. The odor activity value (OAV) indicated that methanethiol, 1-octen-3-ol and 2-heptanone were the key flavor chemicals in raw meat. Hexanal, propionaldehyde, octanal, nonanal, decanal, 1-octen-3-ol and 2-heptanone were key to the flavor of fried meat. Pentanal, decanal, methyl mercap-tan and 1-octen-3-ol contributed greatly to the overall flavor of the roasted meat. Nonanal, methylmercaptan and 1-octene-3-ol had strong effects on the flavor of the steamed silver carp meat.

## 1. Introduction

Silver carp (*Hypophthalmichthys molitrix*) is one of the most economically important freshwater fish species in Asia, Europe, Africa and North America [1]. It is characterized by a wide distribution, high-efficiency cultivation and fine meat quality [2]. China is a populous country. In an effort to diversify food sources [3], silver carp cultivation is being prioritized as the species is one of four major freshwater fishes. In 2021 and 2022, 3,836,600 and 3,713,900 tons of silver carp were produced, accounting for about 14.53% and 15.02% of the total production of freshwater fishes in China, respectively [4,5]. According to traditional Chinese cooking processes, silver carp is usually fried, roasted or steamed, and it is rarely eaten uncooked. Silver carp is rich in protein and other nutrients [6]. Protein can easily be denatured during high-temperature processing. Frying, roasting and steaming are high-temperature processes, and so it is necessary to investigate the effects of cooking processes on the protein nutritional values and flavor of silver carp.

The cooking of fish not only kills pathogenic microorganisms and improves food safety, but also endows the product with superior color and flavor [7]. Different cooking methods have different effects on the nutritional properties and flavor of fish [8]. Recent investigations have found that deep-frying grass carp (*Ctenopharyngodon idellus*) at 1400 W (180 °C) for 2 min and for 2 to 4 min leads to the formation of 13 and 4 volatile compounds, respectively, while also improving the texture and flavor [9]. Steaming and frying increased the dry base protein content of sturgeon (*Acipenser sinensis*) meat by 10.37% and 26.27%, respectively, while the presence of aldehyde compounds such as (E)-2-Octenal, 2-Undecenal and pentadecanal was increased, and the contents of alcohol compounds were reduced [10]. Gradual steaming preserves the juiciness of largemouth bass (*Micropterus salmoides*) meat, reduces toughness as well as lipid and protein oxidation, and adds flavor [11]. Aero-frying causes more severe protein oxidation in scallop (*Patinopecten yessoensis*) meat than baking at the same temperature does [12]. However, according to the literature review, there are few studies on the effects of different cooking methods that can be used to boost the presence of silver carp (*Hypophthalmichthys molitrix*) nutritional value and flavor substances.

Flavor is related to the intuitive perceptions experienced during consumption [13]. The analysis of the effects of cooking methods on the flavor of silver carp will not only help cooks in preparing favored dishes but will also help to clarify the factors contributing to flavor formation. According to An et al. [14], the content of aldehydes in silver carp meat is high, with hexanal, 1-octen-3-ol, 1-octen-3-one, unsaturated aldehydes and sulfur-containing compounds showing strong aromatic properties. However, there are few studies on the effects of different cooking methods on the flavor of silver carp. In order to evaluate the effects of cooking methods on the protein nutrition and flavor of silver carp, three widely adopted cooking treatments (frying, roasting, and steaming) were used to process the meat, and the protein nutritional values and flavor characteristics of the meats were compared. This evaluation of the effects of cooking processes on the protein nutritional values of silver carp will not only guide cooks and fish-processing enterprises to choose a suitable method, which can prevent protein nutrition from being lost, but also remind consumers to choose appropriately cooked products.

## 2. Materials and Methods

### 2.1. Materials and Reagents

Thirty-six silver carps (*Hypophthalmichthys molitrix*) were used, weighing 1800.0 ± 50.0 g and, with body length 44.0 ± 3.0 cm. They were purchased from Chengdu Daiyuan Fishery Co., Ltd. (Chengdu, China). The silver carps were allowed to recuperate in a temporary storage pool with running water (20.0 ± 2.0 °C) for 24 h before slaughter. The dissolved oxygen content of the water was 5 mg/L, and the recuperation density was 32 kg/m^3^. After recuperation, the silver carp were slaughtered in accordance with national standards [15]. We cut off the dorsal muscles of the fish after slaughter, and these were packed and transported to our laboratory on ice within 4 h.

Solid sodium hydroxide, concentrated hydrochloric acid, petroleum ether, copper sulfate pentahydrate, potassium sulfate, sulfuric acid, boric acid, magnesium acetate tetrahydrate, sodium citrate and ninhydrin were all analytically pure and purchased from Chengdu Jinshan Chemical Reagent Co Ltd. (Chengdu, China); the water used for the experiments was ultrapure water derived from a Sartorius ariummini ultrapure water machine (Sartorius Group, Germany).

### 2.2. Cooking Treatment for Silver Carp Meat

According to Peng et al. [16], frying, roasting and steaming were chosen as the cooking methods with which to evaluate the effects of cooking processes on protein nutritional values and the volatile flavor substances of silver carp. The dorsal muscles of the silver carp were cut into 3 × 3 × 1 cm (length × wideness × thickness) pieces, weighing 10.0 ± 1.0 g. In total, 16 pieces (160 g) of meat from three carps were used for the frying, roasting and steaming treatments, with three repetitions in each kind of treatment, and the untreated meat was taken as the control.

Frying processing: After the oil in an induction cooker (Midea MC-CL35W7-001, Guangzhou, China) had reached a temperature of 170.0 ± 5.0 °C, the silver carp pieces were placed in it and fried for 6 min (30 s on each side; the internal temperature was controlled at 70.0 ± 5.0 °C). The residual grease on the meat’s surface was removed with oil-absorbing paper (Jinjiang Hengan Xinxiang Printing paper products Co., LTD., Jinjiang, China).

Roasting processing: The silver carp meat was coated with oil (4.0 ± 0.5 g) and roasted at 180.0 ± 5.0 °C in a K35FK602 electric oven (Zhejiang Supor Co., Ltd., Hangzhou, China) for 10.0 ± 0.5 min (the internal temperature was controlled at 70.0 ± 5.0 °C). The residual oil on the surface was removed with oil-absorbing paper (Jinjiang Hengan Xinxiang Printing paper products Co., Ltd., Jinjiang, China) after the roasting.

Steaming processing: After heating the water in an induction cooker (Midea MC-CL35W7-001, Guangzhou, China) to boiling temperature (98.4 ± 0.6 °C), the silver carp meat was placed on a steaming board and steamed for 10.0 ± 0.5 min (the internal temperature was controlled at 70.0 ± 5.0 °C). We then removed the water from the surface of the steamed meat with filter paper (Fushun City Minsheng filter paper factory, Fushun, China).

### 2.3. Determination of Chemical Composition

Working according to the National Standard for Food Safety of China, the ash content of the silver carp meat was determined using the total ash determination method described in GB 5009.4-2016 [17]. Moisture content was determined using the direct drying method described in GB 5009.3-2016 [18]. The protein content was determined using the Kjeldahl method described in GB 5009.5-2016 [19]. The fat content was determined with Soxhlet extraction, as described in GB 5009.6-2016 [20]. Triplicate experiments were performed for each sample, and the average values were used.

### 2.4. Determination of Amino Acids

The amino acids of the samples were determined according to the methods of Jin et al. [21] and Zhang et al. [22] with slight modifications. In total, 3 g meat pieces were put into a hydrolysis tube, and we added 15 mL of 6 mol/L hydrochloric acid solution; these were then frozen for 5 min, evacuated to 0 Pa, and then filled with nitrogen, before being sealed and hydrolyzed in an oven at 110 °C for 22 h. After cooling to room temperature (25.0 ± 2.0 °C), the hydrolysate was filtered into a 50 mL volumetric flask and fixed with deionized water. Then, 1.0 mL of the filtrate was pipetted into a concentration flask and dried under reduced pressure on a rotary evaporator (RV 3V, IKA, Stauffen, Germany) at 47.0 ± 2.5 °C. The dried residue was dissolved in 1 mL of deionized water. It was then dried again under reduced pressure and evaporated. The sample was dissolved in 1.0 mL of pH 2.2 sodium citrate buffer solution. It was then mixed and passed through a 0.22 μm filter membrane (Tianjin Jinteng experimental equipment Co., Ltd., Tianjin, China) before undergoing determination. An amino acid analyzer (Model L-8900, Hitachi (China) Co., Ltd., Beijing, China) was used to analyze the samples. The analysis conditions in the stationary phase and separation column were as follows: the cation exchange column had a particle size of 3 μm (internal diameter 4.6 mm × 60 mm), and the separation column’s temperature was 57 °C. The detection wavelength was 570 nm and 440 nm; the mobile phase was sodium citrate buffer (pH = 2.2), the flow rate was 0.4 mL/min, and the injection volume was 20 μL. The ninhydrin reaction solution had a flow rate of 0.35 mL/min and the reaction unit temperature was 135 °C.

### 2.5. Nutritional Value Analysis of Silver Carp Meat Protein

The amino acid score (AAS), essential amino acid index (EAAI) and protein efficiency ratio (PER) of the silver carp meat were calculated according to the human amino acid requirement model proposed by the World Health Organization [23,24].

(1) $$AAS=Min\left\{{\left(\frac{aa}{AA}\right)}_{1},\cdots ,{\left(\frac{aa}{AA}\right)}_{k}\right\}$$

(2) $$EAAI=\sqrt[{k}]{{\left(\frac{aa}{AA}\right)}_{1}\times \cdots \times {\left(\frac{aa}{AA}\right)}_{k}}$$

(3) PER=−0.468+0.454×LEU−0.105×Tyr

*AA* is the amino acid content of the reference protein; *aa* is the detected amino acid content in the proteins; and *k* is the number of amino acid types.

### 2.6. Determination of Flavor Substances in Silver Carp Meat

The process of determining flavor substances in fish was performed with reference to the methods of Zhang et al. [25], Peng, Zhang, Bai, Chen, Qian, Xiao and Zhang [16] and Zhang et al. [26], with slight modifications. The sample was extracted via headspace solid-phase microextraction using a PAL RSI multifunctional gas chromatograph autosampler (CTC Analytics AG, Zwingen, Switzerland) under the following conditions: heating chamber temperature of 75 °C, rotational speed of 250 r/min, equilibration for 20 min, aging at 260 °C for 5 min, insertion of a 50/30 DVB/CAR/PDMS extraction head (Supelco, Bellefonte, PA, USA) for 20 min, and resolution for 5 min. GC conditions: GC separation was performed on a DB-WAX UI (30 m × 0.25 mm × 0.25 μm) capillary GC column (Agilent, Santa Clara City, CA, USA) with helium as the carrier gas. This procedure had a flow rate of 1.0 mL/min and an inlet temperature of 250 °C. The ramp-up procedure began at 50 °C and was held for 3.0 min. The temperature was increased to 100 °C at 2.5 °C/min and held for 3 min. This was again increased to 150 °C at 3 °C/min and held for 3 min, and then increased to 250 °C at 15 °C/min and held for 5 min. MS conditions: an electron ionization source (EI) with an electron energy of 70 eV, an ion source temperature of 230 °C, a quadrupole temperature of 150 °C, a detector voltage of 350 V, and a mass scan range (*m*/*z*) of 40– 400 amu. The data obtained were searched using the instrument’s NIST 2017 spectral library data. After this, we performed manual interpretation of the spectra and made comparisons with the reference literature in order to select substances with a match of over 80%. The total ion flux chromatogram was quantified using an internal standard (2 g/L 2,4,6-trimethylpyridine added at 1 μL per sample) and relative peak area, and the results are expressed as 2,4,6-trimethylpyridine equivalents.

### 2.7. OAV Analysis of Flavour Substance Activity Values in Fish

OAV is the ratio of the absolute content of the aroma compound to its flavor threshold, which is used to evaluate the contribution of the compound to the overall flavor of the sample. The higher the OAV value is, the greater the contribution of the compound to the overall aroma of the sample will be [27].

### 2.8. Data Analysis

Analysis of variance was performed using IBM SPSS Statistics 24.0 (International Business Machines Corporation, New York, NY, USA). Results under a significance level of 5% were considered significance (*p* < 0.05).

## 3. Results and Discussion

### 3.1. Effects of Cooking Processing on the Chemical Composition of Silver Carp

To evaluate the effects of cooking processing on the chemical composition of the silver carp meat, the contents of moisture, protein, ash and fat were determined, and the protein, ash and fat contents of the silver carp meat were expressed on a dry basis (Table 1). The data in Table 1 indicate that the moisture content of the raw fish meat (80.80%) was significantly (*p* < 0.05) higher than that of steamed meat (74.93%), which was significantly (*p* < 0.05) higher than that of the roasted (70.70%) and fried (67.06%) samples. Overall, the moisture content of the fried meat was the lowest compared to the other types. The protein content of the fried sample was the highest compared to the other three; the overall ranking of protein content is fried > roasted > raw > steamed. The ash and the fat contents of the four samples showed similar significant (*p* < 0.05) differences, with an overall ranking of fried > roasted > steamed > raw (the reverse of the moisture content ranking). These results suggest that frying, roasting and steaming are conducive to increasing the contents of ash and fat in cooked silver carp meat, while frying and roasting seem able to increase the protein content.

The moisture contents of the cooked meats were all significantly (*p* < 0.05) lower than those of the raw meat, and similar results have been reported when cooking *Polypterus bichir* meat [28]. Frying, roasting and steaming are high-temperature processes; this causes the denaturation of protein, causing the moisture-holding ability of the meat to decrease [29,30]. This may be why cooking resulted in the moisture contents of the samples significantly (*p* < 0.05) decreasing compared to those of the raw meat. According to Section 2.2, the temperatures involved in frying (170 °C) and roasting (180 °C) are higher than those involved in steaming (100 °C). This higher processing temperature may lead to more of the moisture bound in the meat tissue being lost. This may be why the dry-basis protein contents in the fried and roasted meat samples were significantly (*p* < 0.05) higher than those in the raw and steamed samples.

Part of the soluble protein content of the meat was lost via drip loss after steaming [31]. This may be why the dry-basis protein content of the steamed meat sample was significantly (*p* < 0.05) lower than that of the raw sample. Frying, roasting and steaming may induce losses of bound water. This may be why the ash contents of the four samples were similar and ranked in a reversed order compared to the moisture content. However, frying, roasting and steaming may induce losses in bound water because oil is added during frying and roasting. Both of these factors may explain the large differences in the fat contents of the four samples, as well as the reversal of their ranking order compared to that of moisture content. Similar phenomena were discovered when cooking ridgetail white prawn (*Exopalaemon carinicauda*) [32].

### 3.2. Effects of Cooking Processing on the Protein Nutrition of Silver Carp Meat

#### 3.2.1. Amino Acid Content

To evaluate the effects of cooking processes on the protein nutritional values of silver carp, the amino acid contents of raw, fried, roasted, and steamed meat were determined (Table 2). The data in Table 2 indicate that the contents of all the amino acids in the fried meat were significantly (*p* < 0.05) higher than those in others. Of the 16 amino acids in the roasted meat, 11 showed no significant (*p* > 0.05) differences from those in the fried sample. Of the 16 amino acids in the steamed sample, 5 were significantly (*p* < 0.05) more abundant than those in the raw sample. The contents of umami-taste-related amino acids (glutamate and aspartate) in the meat samples could be ranked as fried > roasted > steamed > raw. Both the TAA and EAA of the fried and roasted meats were significantly (*p* < 0.05) higher than those of the steamed and raw meat, while the EAAR values of the four meat samples were not significantly (*p* > 0.05) different. These results further suggest that the protein contents of the fried and roasted meat samples were significantly (*p* < 0.05) higher than those of the steamed and raw meat and that heat-processing could cause the amino acids in the meat to dissolve.

Frying and roasting can lead to the formation of a hard protective layer on the surface of the meat [33]. This hard layer can prevent the loss of silver carp meat proteins during cooking. This may be why the contents of all the amino acids in the frying-treated sample were significantly (*p* < 0.05) higher than those in the others, and explains why the results for 11 of the 16 amino acids in the roasted meat showed no significant (*p* > 0.05) differences from those in the fried sample. The contents of umami-taste-related amino acids (glutamate and aspartate) in the meat samples demonstrate that frying, roasting and steaming are conducive to improving the umami taste of silver carp meat, which can improve the overall flavor and enhance the nutritional intake of the elderly and sickly [34,35]. The temperatures of the frying and roasting treatments were higher than those used in steaming; this may have caused a greater denaturation of protein in the silver carp meat than that seen in steaming, thus resulting in the protein in the silver carp being more easily hydrolyzed into amino acids. The frying, roasting, and steaming processes might not decrease the contents of essential amino acids in the meat. This may be why the TAA and EAA values of the fried and roasted meat samples were significantly (*p* < 0.05) higher than those of the steamed and raw meat samples, but why the EAAR values of the four samples showed no significant (*p* > 0.05) differences.

#### 3.2.2. Protein Nutrition Indexes

To evaluate the effects of cooking processes on the protein nutritional values of silver carp meat, the AAS for children and adults, the EAAI for children and adults, and the PER of the meat samples were compared (Table 2). The data in Table 2 indicate that the AAS for child and adult values of the fried and roasted silver carp meat showed no significant (*p* > 0.05) differences. Conversely, the AAS for child and adult values of the fried and roasted meat were significantly (*p* < 0.05) higher than those of the steamed and raw samples. There was no significant (*p* > 0.05) difference between the AAS for child and adult values of the steamed and raw samples. A similar significant difference was shown for the EAAI and PER values of the four samples, but the EAAI for the adult value of the steamed meat sample was significantly higher (*p* < 0.05) than that of the raw one. These results suggest that frying and roasting can increase the protein nutrition value and potency of silver carp meat.

AAS is the ratio of the first limited amino acids of the sample to the corresponding amino acids in the protein model, EAAI is the ratio of all the amino acids in the sample to the corresponding amino acids in the protein model, and PER represents the protein absorption efficiency [36]. The AAS for child and adult, the EAAI for child and adult, and the PER of the fried and roasted samples were significantly (*p* < 0.05) higher than those of the raw and steamed samples. This indicates that frying and roasting processing increased the protein nutritional value of the silver carp meat. According to the PER values, both these processes can also increase the absorption ratio of silver carp meat protein. They also increased the TAA of the silver carp meat, while the contents of most of the amino acids in the fried and roasted meat were higher than those of the steamed and raw meat (Table 2). These factors may explain the lack of significant (*p* > 0.05) difference in the AAS, EAAI and PER values of the fried and roasted meat samples compared to those of the steamed and raw samples.

### 3.3. Effects of Cooking Processes on Flavor Substances of the Silver Carp Meat

Since the flavor substances are the major contributions of the overall aroma of the product [16], in order to clarify which kind of chemicals produced by frying, roasting and steaming, the volatile flavor compounds in silver carp meat treated with different cooking methods were determined. Table 3, Table 4 and Table 5 show the relative and absolute contents of the volatile flavor compounds in silver carp meat treated with different cooking methods. As can be seen from Table 3, the volatile compounds in the silver carp meat mainly comprised aldehydes, hydrocarbons, alcohols, esters and others.

There were 32 volatile flavor compounds in raw silver carp meat, including 4 aldehydes (2.76%), 8 hydrocarbons (10.55%), 4 alcohols (13.44%), 6 ketones (7.98%), 3 esters (2.24%) and 7 others (15.02). Among these compounds, 2,4-pentanediol (6.44%), n-hexanol (3.03%), 2,2,3-trimethyl-cyclobutanone (4.25%) and 2,6,11-trimethyl-dodecane (4.16%) were present at a relatively high level.

The volatile flavor compounds in the fried silver carp meat were mainly alcohols (two species), which was 27.84% of the content, followed by ketones (seven species) and aldehydes (nine species), with contents of 12.51% and 11.57%, respectively. Compared to the raw silver carp meat, the fried meat had three more volatile compound species and a 12.05% greater content of flavor chemicals (Table 3). The contents of five aldehydes were increased by 8.81%; these included 2-methylpentanal (1.18%), nonanal dimethyl acetal (0.24%), octanal (0.26%), nonanal (0.34%) and heptanal (1.39%). We saw a decrease in the number of alcohol species, but their overall content increased by 14.4%. One ketone species was added, and the overall content of ketones increased by 4.53%, while the contents of hydrocarbons, esters and others decreased.

The volatile flavor substances in the roasted silver carp meat were mainly alcohols (21.76%) that underwent a relative increase of 8.32% compared to the content of the raw meat, the main volatile compounds being n-hexanol (9.37%), 1-penten-3-ol (4.80%) and 1-octen-3-ol (3.93%). All of the volatile compounds detected were reduced in terms of types and relative content, with the exception of alcohols, which were increased in type and relative content, but with a 12.84% reduction in total content compared to raw meat.

The volatile flavor substances in the steamed silver carp meat were mainly aldehydes (23.5%), which showed a 20.74% increase in their relative content compared to the raw meat; the main flavor chemical that was increased was hexanal (17.93%). Compared to raw meat, all other flavor chemicals decreased in relative content. The exception was esters, which were increased by 0.96%.

#### 3.3.1. Aldehydes in Silver Carp Meat

The data in Table 3 indicate that the contents of aldehydes in the silver carp meat can be ranked as fried > steamed > roasted > raw. Compared to raw silver carp meat (Table 4), the contents of propionaldehyde, 2-methylpropionaldehyde, pentanal and hexanal in the fried meat increased, while new compounds (2-methylpentanal, nonanal dimethyl acetal, octanal, nonanal and decanal) were detected in the fried meat. Propionaldehyde and 2-methylpropionaldehyde were not detected in the roasted or steamed meat, while hexanal was not detected in only the roasted meat. The contents of both pentanal and hexanal were increased in the steamed silver carp meat compared to the raw meat, while three new substances (heptanal, nonanal and decanal) were detected. Decanal was only detected in the fried, roasted and steamed meat samples.

Aldehydes are the important contributors to meat flavor and are mainly derived from the oxidative degradation of lipids in meat [37]. Aldehydes have a low threshold for producing fruity, grassy and other odor characteristics, and they contribute to many of the characteristic flavors of fish meat [38]. Heptanal, nonanal, octanal and decanal, which have a fatty flavor, were detected in the fried, roasted and steamed meat, which was most likely due to lipid oxidation [39]. Processing temperature and time are the key factors influencing the oxidation of polyunsaturated fatty acids in meat [40]. The temperatures and times involved in frying, roasting, and steaming were different. The different heat-processing conditions may have generated differences in presence of flavor substances within the cooked meat.

#### 3.3.2. Hydrocarbons in Silver Carp Meat

Hydrocarbons are mainly formed via the oxidation of lipids in meat, and they have a high threshold value. As shown in Table 3, the contents of hydrocarbons in the silver carp meat can be ranked as roasted > fried > raw > steamed. The chemicals 4,5-dimethyl-octane and 3,5,5-trimethyl-2-hexene were detected in all the meat samples, but the contents of 4,5-dimethyloctane, 3,5,5-trimethyl-2-hexene, pentadecane and 3,7-dimethyl-nonane were increased in the fried and roasted compared to the raw meat. The flavor chemical 2,4-dimethyl-decane was only detected in the fried meat, and 3,3-dimethyl-hexane was detected in both the roasted and the steamed meat.

Hydrocarbons are formed via the alkoxy radical homolysis of fatty acids [41]. Hydrocarbon compounds include alkanes, olefins and aromatic hydrocarbon substances. Saturated hydrocarbon compounds have a high threshold and generally have little effect on the flavor of fish meat [8]. Unsaturated hydrocarbons have a relatively low threshold and contribute to fish flavor. Under specific conditions, hydrocarbon compounds can generate flavor substances such as aldehydes, ketones and alcohols, thus playing a potential role in flavor formation in silver carp [42]. The temperatures used for frying, roasting, and steaming the silver carp meat were different. The different heating processes may have caused the differences in the flavor substances within the cooked silver carp meat.

#### 3.3.3. Alcohols in Silver Carp Meat

Alcohols have a high flavor threshold, and therefore contribute less to the overall flavor of silver carp meat [43]. As can be seen in Table 3, the content of alcohols in the silver carp meat can be ranked as fried > roasted > steamed > raw. Hexanol and 1-octen-3-ol were detected when the silver carp meat was fried, roasted and steamed. Two new alcohols (2-ethylcyclobutanol and 1-penten-3-ol) were detected in the roasted meat, and 1-pentanol was only detected in the steamed meat. These new compounds may have been converted from 2,4-pentanediol when the silver carp meat was processed at a high temperature.

Alcohols usually have a pleasant fruity and aromatic flavor. The flavor chemicals in the silver carp meat that are alcohols mainly include fatty alcohols, such as saturated and unsaturated fatty alcohols, which are mainly produced via the oxidation of unsaturated fatty acids during the cooking processing [44]. The higher thresholds of saturated alcohols mean they contribute less to the flavor of the cooked meat, but the lower thresholds for unsaturated alcohols mean they contribute more, especially in terms of metallic, mushroom-like flavors. High-temperature processing induced the degradation of linoleic acid hydroperoxides in the meat [45]. This may be why the content of 1-octen-3-ol in the silver carp meat was increased after cooking.

#### 3.3.4. Ketones in Silver Carp Meat

A total of 11 ketones were detected in the silver carp meat (Table 3). The content of ketones in the silver carp meat could be ranked as fried > roasted > raw > steamed. The chemical 2,3-pentanedione was only detected in cooked meat samples. The chemical 5-hydroxy-2,7-dimethyl-4-octanone was only detected in fried meat, while 3,5-octadiene 2-one was only detected in the roasted meat, and 2-butanone was only detected in the steamed meat.

Ketones are produced via the thermal oxidative degradation of unsaturated fatty acids or amino acids, which mainly produce eucalyptus, fruit and fatty flavors [46]. However, the flavor thresholds of ketones are much higher than those of aldehydes. The high thresholds of ketones mean that they contribute little to the overall flavor of silver carp meat, but can reduce its fishy flavor [47]. Therefore, the higher contents of ketones in the fried and roasted meats suggest that frying and roasting reduces the fishy flavor of this meat.

#### 3.3.5. Esters in Silver Carp Meat

The contents of esters in the silver carp meat can be ranked as fried > steamed > raw > roasted (Table 3). The chemical vinyl hexanoate was only detected in fried, roasted and steamed silver carp meat. The contents of methyl acetate were increased when the meats were fried and roasted. Compared to raw meat, dipyrocetyl was only increased in the steamed meat, while the isoamyl nitrite content increased in the fired meat but decreased in the steamed meat.

Esters are produced via the lipid metabolism or esterification of alcohols and carboxylic acids, and the thresholds of ester compounds are usually low. Short-chain ester compounds generally have a pleasant fruity sweetness, and long-chain ester compounds have a greasy taste [48]. The higher contents of esters in the fried silver carp indicate that frying can impart more flavor to fish products.

#### 3.3.6. Other Compounds in Silver Carp Meat

The table below shows that the contents of other compounds can be ranked as raw > fried > baked > steamed, with dimethylamine and octamethylcyclotetrasiloxane being detected only in raw meat, and the new compound acrylamide only detected in fried meat. The total contents of the other compounds decreased as a result of all the cooking methods. This was probably due to the conversion of this group of compounds into other substances via heating.

Acrylamide is a glycosylation product produced in the late stage of the Maillard reaction in the hot processing of meat, and it is generally common in fried foods [49]. Dimethylamine is only present in raw silver carp because it decomposes into CO and other hydrocarbons during high-temperature heating [50]. The other compounds listed in Table 4 had higher thresholds, and had no significant effects on the overall flavor of silver carp.

#### 3.3.7. OAV Values of the Flavor Compounds

The contributions of volatile compounds to the overall aroma of the product are not only determined by their concentration, but also by their odor threshold. Thus, the OAV values of the flavor chemicals were calculated [51]. The data in Table 5 show that there are six key compounds in raw meat, nine key compounds in fried meat, six key compounds in roasted meat and seven key compounds in steamed meat. In the raw meat, methanethiol, 1-octen-3-ol and 2-heptanone were the main compounds, all with OAV > 10, indicating that these three compounds had a strong influence on the odor of the raw meat.

In fried silver carp meat, the OVA value of hexanal was more than 100, indicating that this chemical was key to the overall flavor of fried sliver carp meat, while the OAV values of propionaldehyde, octanal, nonanal, decanal, 1-octen-3-ol and 2-heptanone were all more than 10, indicating that these six compounds were the second most important chemicals to the flavor of the fried meat.

In the roasted silver carp meat, the OAV values of pentanal, decanal, methyl mercaptan and 1-octen-3-ol were all more than 10, indicating that these four compounds contributed significantly to the overall flavor of roasted meat. The OAV values of 1-pentene-3-alcohol and n-hexanol were greater than 1, and so these two compounds were also found to contribute to the flavor of roasted meat.

In steamed silver carp, the OAV value of hexanal was greater than 200, indicating that it had a great effect on the overall flavor, bringing flavors of fat and apple to the meat. The OAV values of nonanal, methylmercaptan and 1-octene-3-ol were all greater than 10, indicating that these three compounds had strong effects on the flavor of silver carp meat.

## 4. Conclusions

Different cooking processes have different effects on the chemical composition of silver carp meat. The moisture contents of the silver carp meat cooked in different ways can be ranked as raw > steamed > roasted > fried, while the order of protein contents is fried > roasted > raw > steamed. The ash and fat contents of the four samples show similar significant (*p* < 0.05) differences and can be ranked as fried > roasted > steamed > raw. In this study, the contents of all amino acids in the fried meat were significantly (*p* < 0.05) higher than those in other meats. The frying, roasting and steaming processes improved the umami taste and protein nutritional values of the silver carp meat. A total of 51 compounds were detected in the fried, roasted and steamed meat. The OAV values indicate that methanethiol, 1-octen-3-ol and 2-heptanone were the key flavor chemicals in raw meat. Hexanal, propionaldehyde, octanal, nonanal, decanal, 1-octen-3-ol and 2-heptanone were key to the flavor of the fried meat. Pentanal, decanal, methyl mercap-tan and 1-octen-3-ol contributed significantly to the overall flavor of the roasted meat. Nonanal, methylmercaptan and 1-octene-3-ol had strong effects on the flavor of the steamed silver carp meat. Even though the effects of frying, roasting and steaming processes on the nutritional value and flavor substances of silver carp meat have been clarified, further investigations into the effects of these processes on the sensory quality, toxic chemicals, and storing stability of the processed products are needed.

## Figures and Tables

**Table 1:** Comparison of the essential nutrients in the meat of silver carp with different cooking treatments.

Category	Moisture/%	Protein Content in Dry Basis/(g/100 g)	Ash Content in Dry Basis/(g/100 g)	Fat Content in Dry Basis/(g/100 g)
Raw	80.80 ± 0.21 ^a^	24.21 ± 1.40 ^c^	4.15 ± 0.03 ^d^	9.25 ± 0.15 ^b^
fried	67.06 ± 2.59 ^d^	30.11 ± 1.01 ^a^	4.53 ± 0.02 ^a^	36.26 ± 0.59 ^a^
Roasted	70.70 ± 0.80 ^c^	26.03 ± 1.75 ^b^	4.26 ± 0.09 ^b^	12.89 ± 0.62 ^c^
Steamed	74.93 ± 0.99 ^b^	22.53 ± 2.63 ^a^	4.19 ± 0.03 ^c^	10.45 ± 0.63 ^d^

The lowercase letters near the numbers represent the significance (*p* < 0.05) of the difference.

**Table 2:** Amino acid contents of the silver carp meat subjected to different cooking methods.

Types of Amino Acids	Raw (mg/g)	Fried (mg/g)	Roasted (mg/g)	Steamed (mg/g)
Phenylalanine	7.86 ± 0.09 ^d^	10.64 ± 033 ^a^	9.89 ± 0.27 ^b^	8.63 ± 0.11 ^c^
Alanine	10.85 ± 0.04 ^b^	15.80 ± 1.13 ^a^	14.48 ± 0.58 ^a^	11.86 ± 0.40 ^b^
Methionine	5.53 ± 0.08 ^b^	7.90 ± 0.32 ^a^	7.37 ± 0.13 ^a^	6.33 ± 0.15 ^b^
Glycine	8.67 ± 0.28 ^c^	14.63 ± 1.06 ^a^	14.42 ± 1.14 ^a^	10.20 ± 0.30 ^b^
Glutamate	26.65 ± 37 ^d^	40.06 ± 2.24 ^a^	35.31 ± 1.36 ^b^	30.47 ± 57 ^c^
Arginine	10.92 ± 0.05 ^b^	16.22 ± 0.83 ^a^	14.90 ± 0.62 ^a^	12.33 ± 0.02 ^b^
Lysine	18.12 ± 0.16 ^b^	25.60 ± 1.77 ^a^	22.95 ± 0.69 ^a^	20.24 ± 0.19 ^b^
Tyrosine	6.74 ± 0.13 ^c^	9.15 ± 0.23 ^a^	8.61 ± 0.21 ^a^	7.52 ± 0.25 ^b^
Leucine	14.60 ± 0.17 ^c^	20.43 ± 1.06 ^a^	18.82 ± 0.40 ^b^	16.18 ± 0.17 ^c^
Proline	6.08 ± 0.06 ^b^	9.52 ± 0.22 ^a^	9.31 ± 0.76 ^a^	7.08 ± 0.60 ^b^
Serine	7.30 ± 0.13 ^b^	10.45 ± 0.57 ^a^	9.73 ± 0.42 ^a^	7.90 ± 0.65 ^b^
Threonine	7.93 ± 0.10 ^b^	11.41 ± 0.93 ^a^	10.69 ± 0.45 ^a^	8.98 ± 0.49 ^b^
Aspartate	18.59 ± 0.13 ^c^	26.62 ± 1.99 ^a^	23.98 ± 1.00 ^ab^	21.16 ± 0.39 ^bc^
Ileucine	9.09 ± 0.21 ^c^	12.92 ± 0.84 ^a^	11.59 ± 0.30 ^b^	10.24 ± 0.11 ^c^
Valine	8.02 ± 0.12 ^d^	11.22 ± 0.49 ^a^	10.34 ± 0.19 ^b^	9.09 ± 0.21 ^c^
Histidine	5.14 ± 0.02 ^b^	7.27 ± 0.49 ^a^	6.64 ± 0.25 ^a^	5.71 ± 0.16 ^b^
TAA	172.08 ± 0.12 ^b^	249.82 ± 14.40 ^a^	229.00 ± 8.76 ^a^	193.87 ± 4.30 ^b^
EAA	71.15 ± 0.74 ^b^	100.11 ± 5.64 ^a^	91.64 ± 2.43 ^a^	79.67 ± 1.00 ^b^
EAAR/%	41.35 ± 0.45 ^a^	40.08 ± 0.05 ^a^	40.03 ± 0.47 ^a^	41.10 ± 0.40 ^a^
AAS (Child)	0.27 ± 0.01 ^b^	0.38 ± 0.03 ^a^	0.35 ± 0.01 ^a^	0.30 ± 0.01 ^b^
AAS (Adult)	0.32 ± 0.01 ^b^	0.46 ± 0.04 ^a^	0.42 ± 0.02 ^a^	0.36 ± 0.01 ^b^
EAAI (Child)	0.34 ± 0.01 ^b^	0.48 ± 0.03 ^a^	0.44 ± 0.01 ^a^	0.39 ± 0.01 ^b^
EAAI (Adult)	0.64 ± 0.01 ^c^	0.90 ± 0.05 ^a^	0.83 ± 0.02 ^a^	0.71 ± 0.01 ^b^
PER	5.46 ± 0.06 ^b^	7.85 ± 0.46 ^a^	7.17 ± 0.16 ^a^	6.09 ± 0.05 ^b^

Note: TAA represents total amino acid content; EAA the essential amino acid content; EAAR is the essential amino acid ratio; AAS (child) represents the amino acid value of the child’s amino acid requirement pattern; AAS (adult) represents the amino acid valence of the adult requirement pattern for amino acid content; EAAI (child) represents the amino acid index of the child’s amino acid requirement pattern; EAAI (adult) is the amino acid index representing the pattern of adult requirements for amino acid content; PER stands for protein potency. The lowercase letters near the numbers represent the significance (*p* < 0.05) of the difference.

**Table 3:** Relative contents of flavor compounds in the silver carp meat treated with different cooking method.

Compound	Raw		Fried		Roasted		Steamed	
	Types	Relative Content (%)	Types	Relative Content (%)	Types	Relative Content (%)	Types	Relative Content (%)
Aldehydes	4	2.76	9	11.57	2	1.46	5	23.5
Hydrocarbons	8	10.55	8	4.57	6	6.98	5	3.22
Alcohols	4	13.44	2	27.84	5	21.76	4	12.96
Ketones	6	7.98	7	12.51	5	3.4	3	5.33
Esters	3	2.24	3	1.83	1	0.49	4	3.2
Others	7	15.02	6	5.72	4	5.06	4	6.79
Total	32	51.99	35	64.04	23	39.15	25	55

**Table 4:** Absolute contents of flavor compounds in chub meat by cooking method.

RT	Molecular Formula	Name	CAS	Thresholds (μg/kg)	Raw	Fried	Roasted	Steamed
					Absolute Content (μg/kg)	Absolute Content (μg/kg)	Absolute Content (μg/kg)	Absolute Content (μg/kg)
Aldehydes							/	/
2.224	C_3_H_6_O	Propionaldehyde	123-38-6	4.5	40.14	69.85	/	/
2.287	C_4_H_8_O	2-Methylpropionaldehyde	78-84-2	ND	23.67	28.85	/	/
2.866	C_6_H_12_O	2-Methylpentanal	123-15-9	ND	/	144.12	/	/
3.485	C_5_H_10_O	Pentanal	110-62-3	8	37.78	54.63	123.11	74.04
4.294	C_11_H_24_O_2_	Nonanal dimethyl acetal	18824-63-0	ND	/	29.16	/	/
5.194	C_6_H_12_O	Hexanal	66-25-1	4.5	35.7	847.12	/	938.62
8.014	C_7_H_14_O	Heptaldehyde	111-71-7	10	/	/	/	53.61
11.876	C_8_H_16_O	Octanal	124-13-0	0.7	/	32.39	/	/
16.379	C_9_H_18_O	Nonanal	124-19-6	3.5	/	41.31	/	140.84
19.535	C_10_H_20_O	Decanal	112-31-2	2.8	/	170.8	68.03	23.61
Hydrocarbons								
1.849	C_5_H_12_	Pentane	109-66-0	ND	49.83	/	179.37	27.93
4.637	C_7_H_14_O	3-tert-Butyloxy-1-propene	1471-04-1	ND	28.61	29.57	/	/
4.901	C_12_H_26_	3,4,5,6-Tetramethyloctane	62185-21-1	ND	100.71	/	/	/
4.925	C_12_H_26_	2,4-Dimethyldecane	2801-84-5	ND	/	51.11	/	/
8.692	C_8_H_18_	3,3-Dimethylhexane	563-16-6	ND	/	/	107.52	79.41
10.559	C_10_H_22_	4,5-Dimethyloctane	15869-96-2	ND	26.87	29.63	257.67	19.63
20.773	C_9_H_18_	3,5,5-Trimethyl-2-hexene	26456-76-8	730	25.21	70.99	85.4	23.36
21.637	C_15_H_32_	Pentadecane	629-62-9	ND	51.21	61.65	111.75	/
24.243	C_8_H_16_	Pentylcyclopropane	2511-91-3	ND	/	29.77	/	17.93
31.943	C_11_H_24_	3,7-Dimethylnonane	17302-32-8	ND	35.97	51.05	172.8	/
31.903	C_15_H_32_	2,6,11-Trimethyldodecane	31295-56-4	ND	207.59	236.82	/	/
Alcohols								
1.967	CH_4_S	Methyl mercaptan	74-93-1	0.02	110.79	/	252.52	95.56
3.011	C_5_H_12_O_2_	2,4-Pentanediol	625-69-4	ND	320.99	/	/	/
5.206	C_6_H_12_O	2-Ethylcyclobutanol	35301-43-0	ND	/	/	226.94	/
7.312	C_5_H_10_O	1-Penten-3-ol	616-25-1	400	/	/	629.23	/
10.546	C_5_H_12_O	1-Pentanol	71-41-0	4000	/	/	/	105.77
14.893	C_6_H_14_O	1-Hexanol	111-27-3	700	150.98	2742.43	1227.35	342.62
19.299	C_8_H_16_O	1-Octen-3-ol	3391-86-4	7	87.12	668.89	515.42	133.77
Ketones								
2.256	C_9_H_18_O	2,2-Dimethyl-3-heptanone	19078-97-8	ND	66.34	/	/	/
2.746	C_4_H_8_O	2-Butanone	78-93-3	50000	/	/	/	109.62
4.523	C_3_H_6_OS	Mercaptoacetone	24653-75-6	ND	61.45	/	/	/
4.682	C_5_H_8_O_2_	2,3-Pentanedione	600-14-6	ND	/	110.54	101	56.38
7.955	C_7_H_14_O	2-Heptanone	110-43-0	2.8	16.65	99.95	/	/
8.953	C_7_H_12_O	2,2,3-Trimethyl-cyclobutanone	1449-49-6	ND	211.62	1010.85	/	/
11.531	C_4_H_8_O_2_	3-Hydroxy-2-butanone	513-86-0	259	16.65	200.24	144.32	112.7
19.682	C_10_H_20_O_2_	5-Hydroxy-2,7-dimethyloct-4-one	6838-51-3	ND	/	29	/	/
21.875	C_8_H_12_O	3,5-Octadiene 2-one	38284-27-4	150	/	/	69.08	/
24.112	C_8_H_12_O	(E,E)-3,5-octadien-2-one	30086-02-3	ND	/	40.1	67.45	/
33.14	C_8_H_14_O	3-Octen-2-one	1669-44-9	ND	25.25	41.33	63.69	/
Esters								
2.437	C_11_H_10_O_6_	Dipyrocetyl	486-79-3	ND	44.21	/	/	65.71
4.347	C_5_H_11_NO_2_	Isoamyl nitrite	110-46-3	ND	30.52	53.3	/	19.36
5.476	C_3_H_6_O_2_	Methyl acetate	79-20-9	ND	36.68	66.16	/	64.96
13.434	C_8_H_14_O_2_	Vinyl hexanoate	3050-69-9	ND	/	105.68	64.71	17.31
Others								
2.886	C_3_H_5_NO	Acrylamide	79-06-1	ND	/	187.14	/	/
3.011	C_2_H_7_N	Dimethylamine	124-40-3	ND	217.59	/	/	/
4.393	C_7_H_8_	Toluene	108-88-3	1550	43.87	29.57	270.16	/
3.534	C_8_H_24_O_4_Si_4_	Octamethylcyclotetrasiloxane	556-67-2	ND	96.72	86.49	79.65	35.78
7.516	C_10_H_30_O_5_Si_5_	Decamethylcyclopentasiloxane	541-02-6	ND	178.89	105.33	106.38	108.66
15.124	C_12_H_36_O_6_Si_6_	Dodecamethylcyclohexane	540-97-6	ND	124.43	255.51	207.08	141.02
23.342	C_8_H_24_O_4_Si_4_	Octamethylcyclotetrasiloxane-	556-67-2	ND	31.79	/	/	/
29.483	C_2_H_8_O_2_Si	Dimethylsilylene glycol	1066-42-8	ND	55.5	35	/	70.77
Total content					2591.33	7846.33	5793.9	2878.97

**Table 5:** OAV values of compounds with different cooking treatments.

Name	Threshold Value (μg/kg)	Flavour Description	Raw	Fry	Roast	Steam
Propionaldehyde	4.5	Dirt, pungent	8.92	15.52	/	/
Pentanal	8	Jam, bread flavor	4.72	6.83	15.39	9.26
Hexanal	4.5	Fatty, apple aroma	7.93	188.25	/	208.58
Heptaldehyde	10	Fishy, harsh odour	/	/	/	5.36
Octanal	0.7	Grassy, fishy smell	/	46.27	/	/
Nonanal	3.5	Fatty, citrusy, floral	/	11.80	/	40.24
Decanal	2.8	Fatty, citrusy	/	61	24.30	8.43
Methyl mercaptan	4	Rotten vegetable hearts and rotten egg smell	27.7	/	63.13	23.89
1-Penten-3-ol	400	Fruity	/	/	1.57	/
1-Pentanol	4000	Fermented fruit flavor, bread flavor	/	/	/	0.026
1-Hexanol	700	Fruity, sweet, wine-like, grassy	0.22	3.92	1.75	0.49
1-Octen-3-ol	7	Mushroom flavor, grass flavour	12.45	95.56	73.63	19.11
2-Heptanone	2.8	Fruity, creamy	16.65	99.95	/	/

## Data Availability

Data is contained within the article.
